# Characterization of Breast Lesions Using a Novel Combined Approach of Ultrasonography and Elastography Prior to Invasive Procedures: Are New Age Modalities Set to Replace the Diagnostic Giant?

**DOI:** 10.7759/cureus.4954

**Published:** 2019-06-20

**Authors:** Sachin Khanduri, Mazhar Khan, Anvisha Shukla, Shahla Khan, Iffat Ali, Zahid S Ahmad, Minal Hamza, Tariq Imam

**Affiliations:** 1 Radiology, Era's Lucknow Medical College and Hospital, Lucknow, IND

**Keywords:** keywords: breast lesions, breast imaging reporting and data system (birads), elastography, strain ratio, elastography score

## Abstract

Objective

The goal of this study was to evaluate the diagnostic yield of B-mode ultrasound and ultrasound elastography used alone and in combination for differentiating breast lesions into benign and malignant.

Materials and methods

Eighty-five patients were investigated with B-mode ultrasonography and elastography and provided a Breast Imaging Reporting, and Data System (BI-RADS) score based on ultrasonography, strain ratio, and elastography score (ES) based on elastography. Each lesion was then evaluated by a combination method, combining BI-RADS with strain ratio and BI-RADS with elastography score. Each modality was assessed for the successful detection and characterization of the lesion and whether combining ultrasonography B-mode imaging with strain elastography improves diagnosis and is reliable enough to replace invasive procedures such as biopsy that have been the mainstay of diagnosis.

Results

Of 85 lesions, 23 lesions (27%) were found to be malignant, and 62 lesions (72.9%) were benign. When used alone, BI-RADS had 100.0% sensitivity, 13% specificity, 50% and 100% positive and negative predictive values (respectively), and 72.9% accuracy. BI-RADS results were then combined with strain ratio (SR) and ES. BI-RADS with SR had 91.3% sensitivity, 95.2% specificity, 87.5% and 96.7% positive and negative predictive values (respectively), and 94.1% accuracy. Similarly, BI-RADS with ES had 91.3% sensitivity, 93.5% specificity, 84.0% and 96.7% positive and negative predictive values, and 92.9% accuracy.

Conclusions

The combination method performs better at diagnosing breast lesions than BI-RADS alone and can be used as an early and preliminary basis for diagnosis and in settings where invasive procedures cannot be performed. Combining strain elastography and BI-RADS also help characterize which lesions are better suited for biopsy, leading to a decline in unnecessary invasive procedures.

## Introduction

Breast cancer is the most frequently diagnosed cancer and the chief cause of cancer deaths among women worldwide. Breast cancer alone accounts for 25% of all cancer cases and 15% of all cancer deaths among women [1]. It is now the most common cancer in Indian women followed by cervical cancer [2].

Mammography and ultrasonography are the diagnostic methods that have shown the highest sensitivity in the detection of breast cancer; however, mammography performed on dense breasts may often yield false-negative results [3]. Ultrasonography is sensitive in the detection of lesions, but the specificity is poor as most solid lesions are benign.

In the light of the need for a new technique to detect breast carcinoma early and accurately, a new modality known as ultrasonic elastography is being increasingly used. Ultrasonographic elastography (sonoelastography) is a noninvasive imaging technique that can be used to depict relative tissue stiffness or displacement (strain) in response to an imparted force [4-5]. Ultrasonographic elastography is based on the premise that there are significant differences in the mechanical properties of tissues that can be detected by applying an external mechanical force [6-7].

The concept of elastography has been around for almost a decade, but its application in breast imaging and further correlation with ultrasound and biopsy warrant further work and research. This study is focused on the application of ultrasonographic elastography in breast lesions, in characterization of lesions into benign and malignant, and also the comparison between ultrasonography, ultrasonography combined with Breast Imaging Reporting and Data System (BI-RADS), elastography, and biopsy to assess the sensitivity and specificity of each modality in detection and characterization of breast lesion successfully. Also, we have attempted to assess whether combining ultrasonography B-mode imaging with strain elastography improves diagnosis and is reliable enough to replace invasive procedures such as a biopsy.

Few studies have been conducted that compare these modalities but, to the best of our knowledge, there has been no exhaustive study that has compared all these modalities and used combined methods for the assessment of breast lesions with a histopathological correlation. Also, no such study has been undertaken in an Indian population and, as already discussed, breast density is identified as an independent and the strongest risk factors for breast cancer-this can be different in different ethnic groups and people from different geographical areas [8].

The breast density of a Western population is greater when compared to an Indian population [8], and mammography on dense breasts can yield false negative results, making this study and its combined approach relevant to a Western population as well. The assessment of the breast and its lesions will be of importance in risk assessment and prevention strategies in various groups. This study is a step in that direction.

## Materials and methods

This prospective study was conducted, after obtaining approval from the local ethics committee, in 85 patients admitted to the Era’s Medical College and Hospital, Lucknow, with clinical concerns between October 2017 and October 2018.

The patients we selected had the existence of a breast lesion as detected by B-mode ultrasonography and who had no history of previous treatments, such as breast surgery, chemotherapy, or radiotherapy.

After consent, patients underwent a radiological workup including B-mode ultrasonography. The BI-RADS category was assigned to each lesion, and strain elastography examinations were performed. All patients then underwent a core biopsy. The results of pathologic examination of the obtained specimen were used as the gold standard for comparison of the results of imaging studies.

Ultrasonography images of the lesion were acquired with a GE Healthcare Voluson P9 with a linear transducer at 9 MHz. Each lesion was evaluated for size, shape, echo pattern, and calcification. These lesions were characterized only on ultrasonography and not on mammography and were classified as BI-RADS 3, 4 or 5. BI-RADS 3 were considered benign while BI-RADS 4 and 5 were considered malignant. BI-RADS 4 was further classified into 4A, 4B, and 4C if the likelihood of malignancy was 2% to 10%, 10% to 50%, and 50% to 95%, respectively.

For evaluating the hardness or stiffness of a lesion, strain elastography was performed using a GE Healthcare Voluson P9 with a linear transducer at 9 MHz. For strain elastography, the compression method described by Itoh et al. was used [9]. The ultrasonography transducer probe was positioned parallel to the breast lesion while the patient was in a supine position. Pressure was applied over the breast tissue resulting in its displacement by 1 mm to 2 mm posteriorly and then coming back to its initial location; elastography images for the same range were acquired. The elastography images were assessed using the elastography scoring (ES) system defined by Itoh et al. [9]. Lesions with scores of 1, 2, and 3 were considered benign, and those with scores of 4 and 5 were considered malignant. For the strain ratio (SR) calculation, a region of interest (ROI) box was placed on the lesion while another ROI box was placed on reference tissue. We used adjacent subcutaneous fat tissue as the reference tissue. The strain value of the reference tissue was compared with that of the lesion for the calculation of SR. For classification via strain elastography, a single cutoff value was used. Lesions with a mean SR value equal to or greater than the cutoff value were considered malignant, and those with an SR value less than the cutoff value were considered benign. For ES classification, the cutoff value was accepted as classification 3; lesions that had an ES of 1, 2 or 3 were regarded as benign, and those with an ES of 4 or 5 were considered malignant.

The approach of this study was to combine ultrasonography and elastography to assess whether we could obtain a better characterization of breast lesions. After combining these methods, the sensitivity, specificity, positive predictive value (PPV), negative predictive value (NPV), and accuracy were calculated to assess the result of the combined approach. Herein we combined BI-RADS and SR, and BI-RADS and ES, respectively. BI-RADS 3 and 5 breast lesion scores were used as such. BI-RADS 4 lesions were subcategorized as 4A, 4B, and 4C according to the suspicion of malignancy. These lesions were categorized again according to the results of SR and ES, and a new BI-RADS category was provided. SR results with a cutoff value of 2.8 was re-categorized as BI-RADS 3 while those above 2.84 were re-categorized as BI-RADS 5. Similarly, lesions classified as ES 1, 2, or 3 were re-classified as BI-RADS 3, and those with an ES of 4 or 5 were re-classified as BI-RADS 5.

For BI-RADS 4 and 5 classified lesions, a biopsy was recommended. The histopathology of all lesions was performed by core biopsy under ultrasonographic guidance. For BI-RADS 3 lesions, although not necessary, histopathology was also obtained from these lesions for research purposes and further characterization.

Statistical analysis

Categorical variables were presented in number and percentage (%), and continuous variables were presented as mean ± standard deviation and median. Qualitative variables were compared using a chi-square test or Fisher’s exact test as appropriate. The area under the receiver operating characteristic (ROC) curves was used to evaluate the predictive ability of a method when using maximum cutoff values. We determined the rates of sensitivity, specificity, PPV, NPV, and accuracy. A P value of < .05 was considered statistically significant. The data analysis was performed using Statistical Package for Social Sciences (SPSS) for Windows, Version 16.0 (SPSS Inc., Chicago, USA).

## Results

We studied 85 patients. The mean patient age was 38.8 ± 11.8 years (range, 19 to 67 years). The mean size of the lesions as calculated according to the longest axis was 45 ± 12.8 mm (range, 20 to 70 mm). The characteristics of the breast lesions are presented in Table 1.

**Table 1:** Characteristics of breast lesions on ultrasonography

Characteristics		Number of lesions (n)	Percent (%)
Patient	Benign	62	72.9
	Malignant	23	27
Shape	Irregular	22	25.9
	Oval	23	27.1
	Round	40	47.1
Echo pattern	Hyperechoic	4	4.7
	Hypoechoic	76	89.4
	Mixed	5	5.9
Calcification	Present	10	11.8
	Absent	68	80
	Present/Absent	7	8.2

The histopathological results of the breast lesions in the study are shown in Table 2.

**Table 2:** Histopathological results of breast lesions

Histopathology	Number of lesions (n)	Percent (%)
Fat necrosis	4	4.7
Fibroadenoma	33	38.8
Fibrocystic changes	1	1.2
Giant phyllodes	3	3.5
Granulomatous mastitis	9	10.6
Hyperplasia	1	1.2
Invasive ductal carcinoma	10	11.8
Invasive lobular carcinoma	7	8.2
Invasive micropapillary carcinoma	1	1.2
Medullary carcinoma	4	4.7
Neuroendocrine carcinoma	1	1.2
Phyllodes	6	7.1
Sclerosing adenosis	5	5.9

Elasticity score

**Figure 1:**
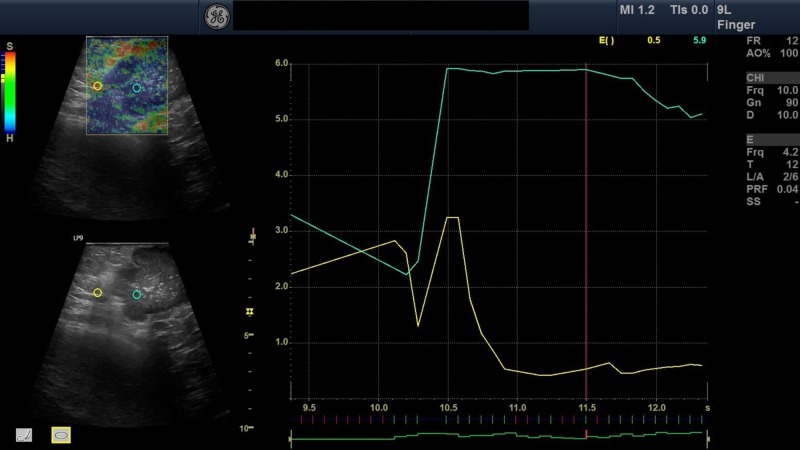
Elastography image of breast lesion A 45-year-old woman presented with a concern of right side breast mass with pain and discharge from the nipple. On palpation, the mass was hard. The mass was in the upper outer quadrant involving two-thirds of the breast parenchyma. B-mode ultrasonography of breast was done followed by strain elastography. On ultrasonography, a large ill-defined lobulated heteroechoic mass was seen with few foci of calcification and increased vascularity. The mass was graded as BI-RADS grade 4C. On elastography, two regions of interests were taken represented by green and yellow circles. The yellow and green region of interest represents normal area and area of mass respectively with their graphic representation (left side). Elastography score of the lesion was 4 and strain ratio of the lesion was 3.9.

**Figure 2:**
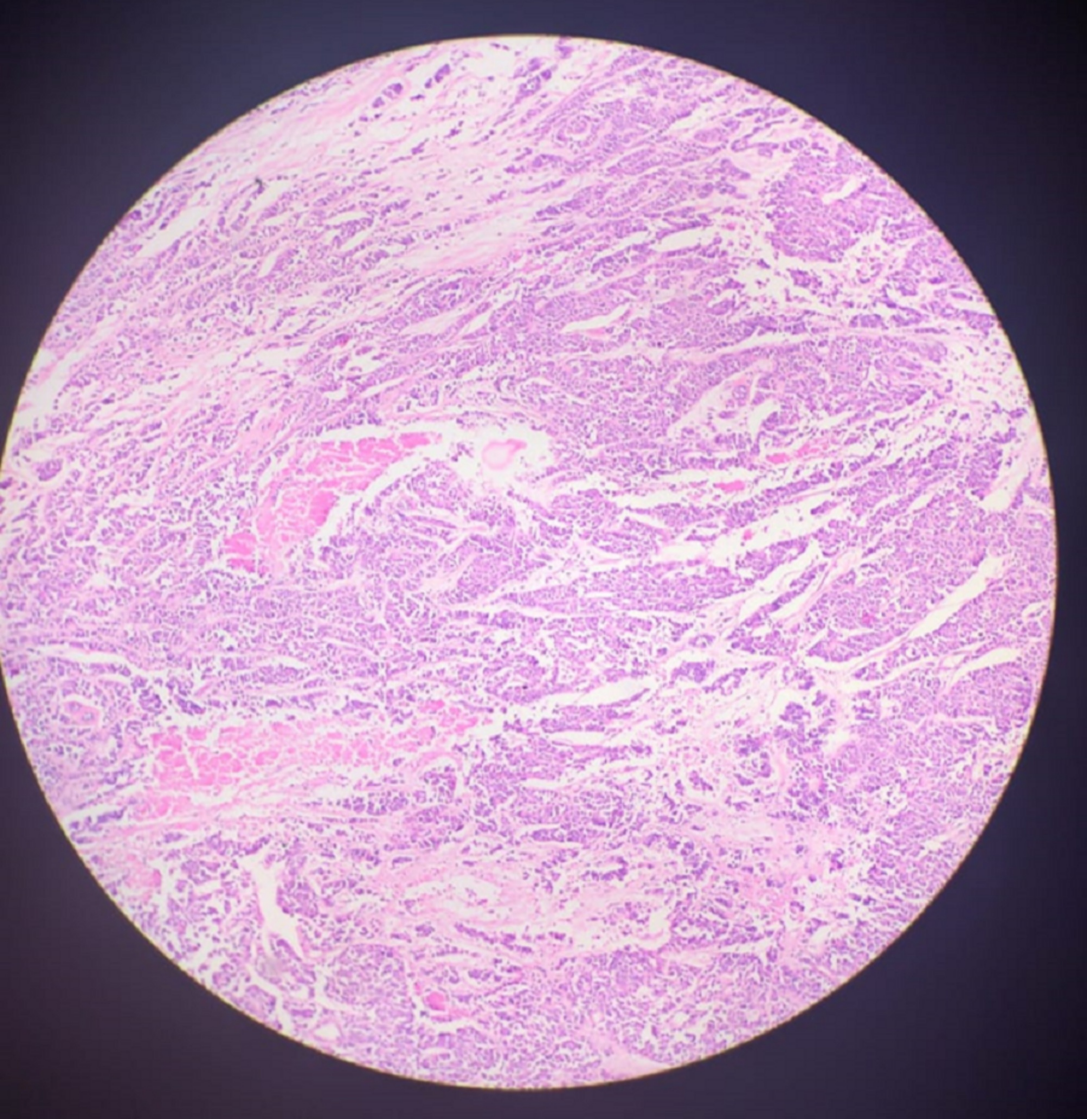
Histopathology slide A section from breast tissue of a 45-year-old woman diagnosed as BI-RADS 4C and had elastography score 4, strain ration 3.9, shows infiltrating atypical cells lying singly in nests and cords. These cells show marked anisonucleosis having pleomorphic hyperchromatic nuclei, prominent nucleoli, coarse chromatin surrounded by pale amphophilic cytoplasm. A few congested blood vessels are also seen. The above findings are suggestive of invasive ductal carcinoma of the breast.

The rates of sensitivity, specificity, PPV, NPV, and accuracy of ES were calculated as 87.0% (20/23), 93.5% (58/62), 83.3% (20/24), 95.1% (58/61), and 91.8% (78/85), respectively. Twenty-four (28.2%) lesions were assessed as ES 4 or 5. Of these lesions, 20 (83.3%) were diagnosed as malignant (Figures 1-2) while four (16.6%) were diagnosed as benign. Four false-positive benign lesions were classified as ES 4. Two of these were diagnosed as giant phyllodes and two as phyllodes tumor upon histopathological investigation. Sixty-one (71.8%) lesions were assessed as ES 1, 2, or 3. Fifty-eight (95.0%) and three (4.9%) of these lesions were histopathologically diagnosed as benign and malignant, respectively. Among three false-negative lesions, one was diagnosed as invasive ductal carcinoma, one as medullary carcinoma, and one as invasive lobular carcinoma.

Strain ratio

**Figure 3:**
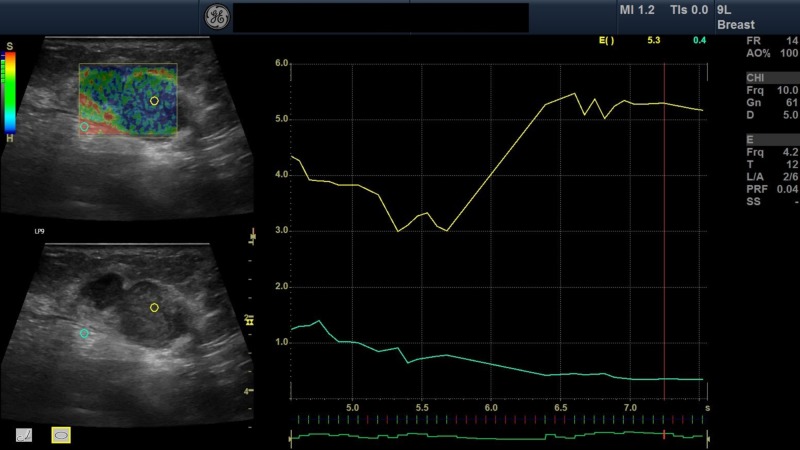
Elastography image of breast lesion A 43-year-old woman presented with concerns of left side breast pain and mass in the lower outer quadrant that was firm in consistency on palpation. The mass was non-mobile with no discharge. On B-mode ultrasonography followed by strain elastography, the mass was well-defined rounded heteroechoic with minimal vascularity and no area of calcification. Mass was graded as BI-RADS grade 3 followed by strain elastography, two regions of interest (green and yellow) were taken. The green and yellow regions of interest represent the normal area and breast mass, respectively, with their graphic representation on the left side. The elastography score of the lesion was 2, and the strain ratio was 1.2.

**Figure 4:**
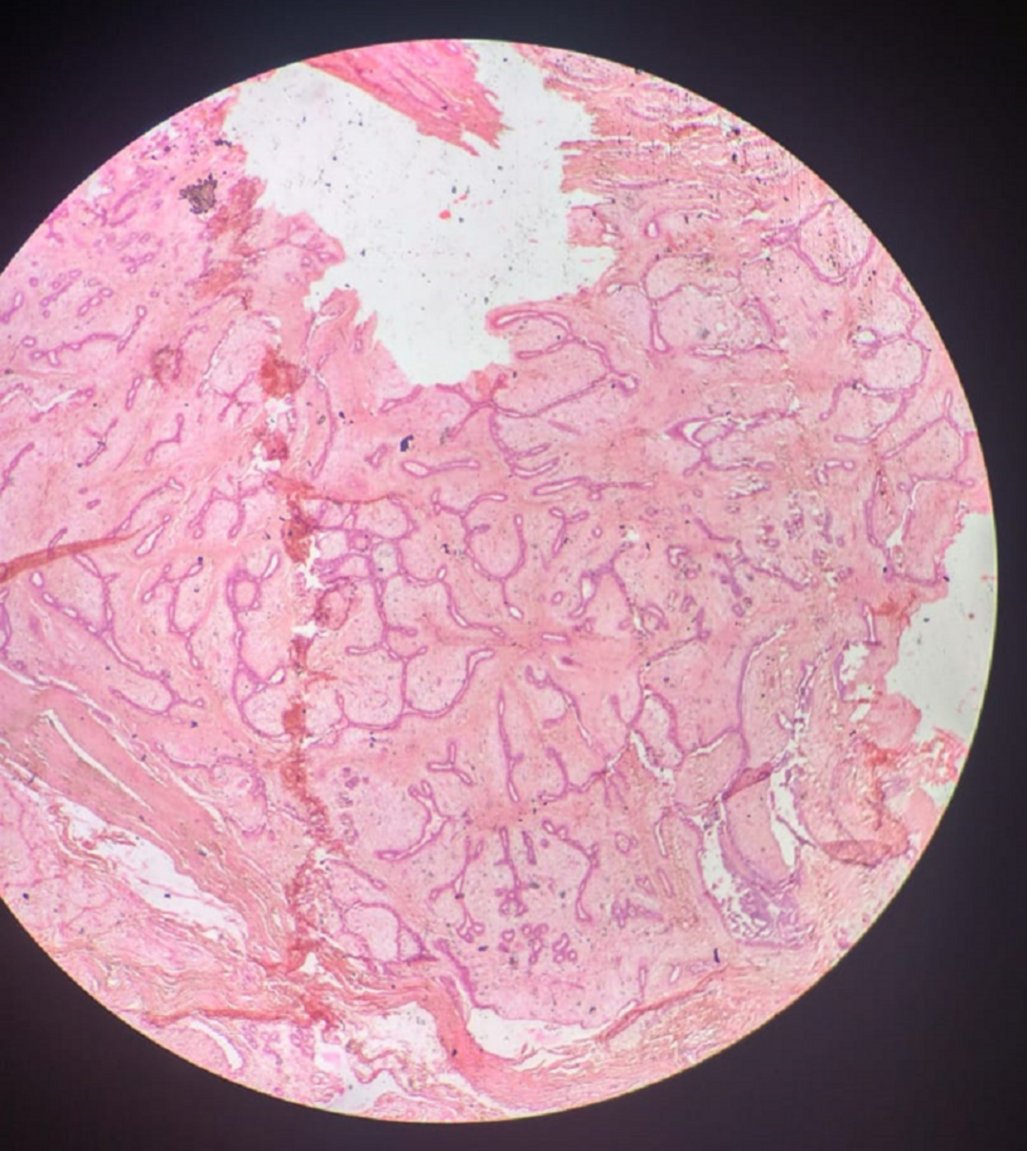
Histopathology slide A section of breast tissue from a 43-year-old woman that was diagnosed with BI-RADS 3 on ultrasonography and had an elastography score of 2 and strain ratio of 1.2 showed both benign glandular as well as hypercellular stromal components. The glands are patent and lined by cuboidal to low columnar epithelium with basally-placed nuclei lying on myoepithelial cell layer. The focal area shows few unremarkable glands compressed by proliferating fibrovascular stroma. The stroma is infiltrated by a mild inflammatory infiltrate composed of lymphocytes, polymorphs and plasma cells. A focus of mature adipose tissue is also seen. The findings are suggestive of benign phyllodes tumor of the breast.

The rates of sensitivity, specificity, PPV, NPV, and accuracy were calculated as 87.0% (20/23), 95.2% (59/62), 87.0% (20/23), 95.2% (59/62), and 92.9% (79/85), respectively. The cutoff value for SR was determined to be 2.85 as this was where maximum sensitivity and specificity were achieved. This was done using the ROC curve. A ROC of SR values was drawn to distinguish between benign and malignant lesions. The area under the ROC value was 0.976; this was found to be statistically significant (P < .001). Twenty-four (28.2%) lesions had SR values greater than 2.85. Of these lesions, 21 (87.5%) were diagnosed as malignant while three (12.5%) were diagnosed as benign (Figures 3-4). One of the false positive lesions were diagnosed as phyllodes and the other two as giant phyllodes. Sixty-one (71.8%) lesions had an SR value less than 2.85. Fifty-nine (97.0%) and two (3.3%) of these lesions were histopathologically diagnosed as benign and malignant, respectively. One of the false negative lesions was diagnosed as invasive ductal carcinomas and the other as invasive lobular carcinoma.

Combination of BI-RADS and strain elastography

When we combined the results of SR and ES with the results of BI-RADS, the sensitivity, specificity, PPV, NPV, accuracy, and area under ROC were affected; the results of this are shown in Table 3. Based on these results, the specificity, PPV, and accuracy of both methods combined with SR and ES increased in comparison with BI-RADS alone. Additionally, the sensitivity, PPV, NPV and accuracy of the combined methods also increased in comparison to SR alone, and the sensitivity, specificity, PPV, NPV, and accuracy of the combined methods increased in comparison to ES alone.

**Table 3:** Data comparing results of BI-RADS, SR, ES alone, and combined BI-RADS+SR and BI-RADS+ES AUROC, area under a receiver operating characteristic curve; BI-RADS, Breast imaging reporting and data system; ES, elastography score; NPV, negative predictive value; PPV, positive predictive value; SR, strain ratio.

Group	Cutoff	AUROC	Sensitivity (%)	Specificity (%)	PPV (%)	NPV (%)	Accuracy (%)
SR	2.8	0.976	87.0(20/23)	95.2(59/62)	87.0(20/23)	95.2(59/62)	92.9(79/85)
ES	Score 3	0.908	87.0(20/23)	93.5(58/62)	83.3(20/24)	95.1(58/61)	91.8(78/85)
BI-RADS	Category 3	0.935	100.0(23/23)	13(3/23)	50 (23/46)	100(3/3)	72.9(62/85)
BI-RADS+ES		0.924	91.3(21/23)	93.5(58/62)	84.0(21/25)	96.7(58/60)	92.9(79/85)
BI-RADS+SR		0.932	91.3(21/23)	95.2(59/62)	87.5(21/24)	96.7 (59/61)	94.1 (80/85)

## Discussion

BI-RADS was developed to be applied to mammography only and does not pertain to other breast imaging techniques. As breast sonography is now a well-established adjunct to mammography and in light of the widespread use of sonography, the American College of Radiology recently developed a BI-RADS lexicon for breast sonography to standardize the characterization of the sonographic lesion [10]. BI-RADS has become an important B-mode ultrasound classification system used to estimate malignant lesions by standardizing breast lesions according to their morphological features. BI-RADS has a high sensitivity rate, but the specificity rate of BI-RADS is still an area of concern. In a similar study performed by Arslan et al. that combined BI-RADS and strain elastography and BI-RADS and ES, an increase was detected in the rates of specificity, PPV, and accuracy compared to BI-RADS alone. These rates were reported as 93%, 91.1%, and 91.3%, respectively, for BI-RADS with SR while the PPV increased to 97.1%, accuracy increased to 92.5%, and specificity showed no change for BI-RADS with ES [11]. In another study performed by Hao et al., where ultrasound elastography and BI-RADS were combined, an increase in specificity, PPV, and accuracy was detected; the rates were 72.9%, 76%, and 82%, respectively [12]. In our study, we found similar results showing an increase in specificity, PPV, and accuracy via both combined methods as compared to BI-RADS alone; rates increased to 93.5% for specificity, 84% for PPV, and 92.9% for accuracy with BI-RADS with ES, while they were 95.2%, 87.5%, and 94.1%, respectively, in the case of BI-RADS with SR. With regards to the combined methods compared with BI-RADS alone, our findings were consistent with other studies [11-13].

Additionally, we also found a significant increase in specificity with BI-RADS + ES as compared to BI-RADS alone. However, with regards to strain ratio and ES alone as opposed to the combination of BI-RADS with each, we found an increase in sensitivity, PPV, and accuracy; the rates were 87.5% and 84.0% for PPV for BI-RADS + SR and BI-RADS + ES, respectively, and 91.3% for sensitivity in both. However, the accuracy only increased in BI-RADS + SR to 94.1%. It remained unchanged in BI-RADS + ES.

Our results demonstrated that BI-RADS combined with elastography provides the largest improvement in accuracy, PPV, and specificity, areas that have been a problem for BI-RADS alone. Based on these results, we conclude that BI-RADS combined with elastography has better diagnostic performance than BI-RADS alone.

We noted that SR and ES both had almost the same diagnostic performance in terms of sensitivity. However, SR provides a much better assessment of larger lesions than ES as larger lesions are the most prone to changes such as degeneration, necrosis, or hemorrhage, affecting the stiffness of the lesions. Zhi et al. described a similar finding in their study [14]. Due to this, these lesions that are supposed to be ES 4 or 5 are often given a lower score, leading us to believe that SR is a better diagnostic tool as compared to ES, particularly for larger lesions.

In general, the rates of false negative and false positive results were reduced when the combination method was used. However, when strain elastography was used alone, false negative results were found in invasive ductal and lobular carcinomas and false-positive results were seen in phyllodes and giant phyllodes. These false negative and false positive results were consistent with other studies and may be due to necrosis, hemorrhage, or the soft nature of the tumors observed in malignant breast lesions and the calcification and fibroblastic proliferation seen in benign breast lesions [9,11,13]. According to Tan et al., phyllodes tumors are classified into benign, borderline, and malignant grade categories on the basis of a constellation of histological parameters leading to significant challenges in accurate and reproducible categorization [15].

Another advantage that the combined method offers is the categorization of a lesion requiring biopsy: if a BI-RADS category 3 lesion shows a hard elasticity representing malignancy, the BI-RADS category can be changed to category 4, leading to earlier diagnosis. The opposite is also true for lesions with a low suspicion of malignancy: BI-RADS category 4A can be converted to BI-RADS category 3, depending upon the elasticity of lesion on ultrasound elastography.

## Conclusions

It can be concluded that the combined use of B-mode ultrasonography and elastography achieved higher accuracy thus avoiding unnecessary invasive diagnostic procedures for categorizing lesions. The combined method is better for diagnosis as opposed to using BI-RADS or elastography alone. Also, BI-RADS 4 lesions can be better categorized via this method. We also found that SR and ES are complimentary. However, in clinical practice, ES is more practical and convenient, while SR is better for differentiating benign and malignant breast masses.
